# Trigeminal Neurosensory Deficit and Patient Reported Outcome Measures: The Effect on Life Satisfaction and Depression Symptoms

**DOI:** 10.1371/journal.pone.0072891

**Published:** 2013-08-29

**Authors:** Yiu Yan Leung, Terence Chak Pui Lee, Samuel Mun Yin Ho, Lim Kwong Cheung

**Affiliations:** 1 Oral and Maxillofacial Surgery, Faculty of Dentistry, The University of Hong Kong, Hong Kong, Hong Kong; 2 Psychology Unit, Faculty of Social Sciences, Hong Kong Baptist University, Hong Kong, Hong Kong; 3 Department of Applied Social Studies, City University of Hong Kong, Hong Kong, Hong Kong; The James Cook University Hospital, United Kingdom

## Abstract

**Objectives:**

To investigate the effect of persistent neurosensory disturbance of the lingual nerve (LN) or inferior alveolar nerve (IAN) on life satisfaction and depression symptoms.

**Methods:**

This study recruited patients with persistent LN or IAN deficit as a consequence of lower third molar surgery for 12 months or more to form the study group. A control group was formed by matching age and gender of recruited subjects in the study group with patients without the neurosensory complications. Life satisfaction was assessed with Satisfaction With Life Scale (SWLS) and depression symptoms were assessed with 20-item Center for Epidemiological Studies Depression scale (CESD-20).

**Results:**

Fourty-eight participants (24 cases and 24 controls) were recruited. Patients with LN or IAN deficits after lower third molar surgery were less satisfied with their lives when compared to the control group (p<0.001). They were presenting with more depression symptoms (p = 0.001). 45.8% of the study group subjects had a CESD-20 score of 16 or above. Older patients presented with more depression symptoms among the subjects with neurosensory disturbance after lower third molar surgery (p = 0.02).

**Conclusions:**

Individuals with permanent trigeminal neurosensory deficit after lower third molar surgery have worse life satisfaction and more depressive symptoms when compared to those who did not suffer from the surgical complication.

## Introduction

Lingual nerve (LN) and inferior alveolar nerve (IAN) injuries deficits affect an individual’s sensation in the tongue or the lower lip region on the affected side. The most common cause of LN or IAN deficits is from lower third molar surgery. These neurosensory deficits and their biological behaviors were well reported in the literature [1]. However, their effects on the affected individuals from the patients’ perspective were not well studied. It was reported the neurosensory deficit have caused psychological distress on the affected individuals, and a portion of these cases involve a litigation process [2].

This is a study investigating the effects of persistent trigeminal nerve deficit after lower third molar surgery on patient reported outcome measures. We are investigating the effect of the trigeminal neurosensory deficit on the patient’s perception on their life satisfaction, as well as the prevalence of depression symptoms.

Life satisfaction refers to a judgmental process, in which individuals assess the quality of their lives on the basis of their own unique set of criteria [3]. It is considered to be a conscious cognitive judgment of a person’s life and it is up to the person’s own criteria of judgment [4]. Satisfaction with Life Scale (SWLS) was developed to assess the life satisfaction of the respondent’s life as a whole [5]. In particular, patients with LN or IAN injury after third molar surgery often suffer from sensory disturbances that are difficult to quantify or measure with an objective scale. However, the impacts of these injuries upon the patient’s quality of life can be reflected by a global measure of his/her life satisfaction such as the SWLS.

Patients with permanent LN or IAN injury after third molar surgery have often been reported to be unhappy or even depressed. The diagnosis of depression is normally made through the use of structured diagnostic interviews based on the Diagnostic and Statistical Manual of Mental Disorder (DSM-IV) [6]. However, the process of the diagnosis is time-consuming and requires special expertise. It is difficult to make a clinical diagnosis of depression after third molar surgery-related nerve injury, which masks the incidence of the event. A screening tool of depressive symptoms may therefore facilitate case-finding and may help clinicians to make appropriate referrals if one with third molar surgery-related nerve injury is suspected to suffer from depression.

The purpose of this study was to investigate the effects of permanent neurosensory deficit after lower third molar surgery on life satisfaction and depressive symptom prevalence of the affected individuals. Patient-related or neurosensory related factors that may affect the life satisfaction and depressive symptoms in patients with neurosensory deficit after lower third molar surgery were also investigated.

## Materials and Methods

### Sampling and Data Collection

This paper is a second part of a study investigating the effects of persistent trigeminal nerve deficit after lower third molar surgery on patient reported outcome measures [7]. Patients with persistent LN and/or IAN deficit for 12 months or more and reviewed in the Discipline of Oral and Maxillofacial Surgery, the Prince Philip Dental Hospital, Hong Kong were enrolled in the Study Group. The inclusion criteria of the Study Group were patients aged 18 years or older, with persistent LN or IAN deficit as a consequence of lower third molar surgery, and the neurosensory deficit confirmed subjective and objective neurosensory assessments. Subjective neurosensory assessment included a rating of their numbness by visualized analog scale (VAS) from 0 (normal) to 10 (most severely affected). Objective neurosensory assessments consisted of three tests: light touch threshold with Von Frey fibres, two-point discriminations and pain threshold. Presences of pain, hyperaesthesia or taste disturbance were also recorded. Neurosensory deficit was defined when subjective numbness VAS was greater than 0, and objective assessments are different from the unaffected side. Those patients whose LN or IAN deficit was not a consequence of lower third molar surgery were excluded. Patients who were treated in the same unit without any neurosensory deficit were recruited sequentially as the Control Group by matching the age (within 2 years) and gender of the sample in the Study Group. The study protocol was approved by the Institutional Review Board of the University of Hong Kong/Hospital Authority Hong Kong West Cluster (Protocol no. UW 11–451). All participants were required to sign consent before being enrolled into the study.

The age, gender and educational background of the participants were recorded. Data of the neurosensory deficit of the Study Group including the affected nerve, duration of the deficit, presence of hyperaesthesia or pain were recorded.

### Instruments

Satisfaction with Life Scale (SWLS) was used to measure the life satisfaction of the study participants [5]. SWLS is a global measure of an individual’s subjective well-being. It has 5 items with a 7-point Likert scale (1 = strongly disagree, 7 = strongly agree). The five questions are (1) In most ways in my life is close to my ideal; (2) The conditions of my life are excellent; (3) I am satisfied with my life; (4) So far I have gotten the important things I want in life; (5) If I could live my life over, I would change almost nothing. The range of the SWLS total score is from 5 to 35, with a higher score indicating an individual being more satisfied with his/her life.

The 20-item Center for Epidemiological Studies Depression scale (CESD-20) was used to measure the depressive symptoms of the participants in the study. CESD-20 is widely used as a screening tool for the depression symptoms in the general population. It measures the frequency of symptoms in the past 7 days and rates on a 4-point scale: 0 for “less than a day”, 1 for “1–2 days”, 2 for “3–4 days” and 3 for “5–7 days”. 4 out of the 20 items are reversely scored. After correction of the reverse scored items and summing the scores, a total score of CESD-20 was calculated, while higher scores indicated greater likelihood of depression. It was suggested a cutoff CESD-20 score of 16 might indicate the need for further assessment of diagnosing clinical depression [8]. In this study we compared participants with high CESD-20 scores (16 or above) to those with low CESD-20 scores (15 or below) in the Study Group.

### Outcome Measures

The primary outcomes of the study were the differences of mean SWLS total score and mean CESD-20 score of the Study Group and the Control Group. The secondary outcomes were the effects of age and gender of the patients, the nerve involved, duration of neurosensory deficit, and the presence of pain or hyperaesthesia of the affected area on the SWLS total score and CESD-20 scores in the Study Group. The patterns of these parameters were also compared between patients with high CESD-20 scores (16 or above) and those with low CESD-20 scores (15 or below) within the Study Group.

### Statistical Analysis

Comparison of the SWLS total scores and CESD-20 scores between the Study/Control Group were performed using paired sample t-test. Statistical analyses were also performed to analyze the difference of the SWLS total scores and CESD-20 scores recorded from patients with various parameters including age and gender of the patients, the nerve involved, duration of neurosensory deficit, and the presence of pain or hyperaesthesia of the affected area using paired sample t-tests. Data were analyzed with the Statistical Package for Social Sciences (SPSS version 19.0 SPSS Inc, Chicago, IL, USA). The 5% probability level was taken as the cut-off for statistical significance.

## Results

Forty-eight patients were recruited in the study. Twenty-four patients (9 males, 15 females) presented with persistent LN (10 patients) or IAN (14 patients) deficit after lower third molar surgery formed the Study Group. The mean age (S.D.) of these patients was 39.6 years (10.8 years). The Control Group included 24 patients without neurosensory deficit and they matched in terms of age and gender with the Study Group. The demographic and neurosensory deficit related data of the two groups are presented in Table 1 and Table 2. None of the recruited subjects reported to be diagnosed with depression.

**Table 1:** Demographic characteristics of the study participants.

	Study Group (n = 24)		Control Group (n = 24)	
	Number	%	Number	%
**Gender**				
Male	9	37.5	9	37.5
Female	15	62.5	15	62.5
**Education**				
Secondary	5	20.8	6	25
Tertiary	19	79.2	18	75
**Affected nerve**				
IAN	14	58.3		
LN	10	41.7		
**Mean Age (S.D)**	39.6 years	(10.8 years)	39.4 years	(10.7 years)
**Mean time of nerve injury (S.D)**	55.8 months	(57.4 months)		

**Table 2:** Neurosensory deficit characteristics of the Study Group participants (n = 24).

	Inferior Alveolar Nerve (n)	Lingual Nerve (n)
**Presence of hyperaesthesia**		
Yes	5	3
No	9	7
**Presence of Pain**		
Yes	1	2
No	13	8
**Taste Disturbance**		
Yes		2
No		8
**Time lapse of nerve injury (S.D.)**	54.3 months (63.2 months)	58 months (51.4 months)
**Mean Numbness in VAS (S.D.)**	4.1 (2.2)	4.0 (2.7)

The mean SWLS total score of the Study Group was 19.4 (S.D. 6.8), which was significantly lower than the score of 27.0 (S.D. 4.5) in the Control Group (p<0.001). It indicated a significantly reduced life satisfaction in patients with persistent neurosensory deficit after lower third molar surgery when compared with individuals who did not have such morbidity. The mean CESD-20 score of the Study Group was 16.4 (S.D. 12.9). It was significantly higher than the Control Group, which was 5.7 (S.D. 6.3) (p = 0.001). It indicated that patients in the Study Group displayed more depressive symptoms than those in the Control Group. 45.8% (11/24) of the patients in the Study Group had a CESD-20 score of 16 or above. It was significantly more than the 8.3% (2/24) of Control Group sample who scored 16 or above in the CESD-20 score (p = 0.003) (Table 3). Of note, most of the participants with CESD-score 16 or above in the Study Group (90.9%, 10/11) scored over 20, indicating the possibility of development into clinical depression.

**Table 3:** Comparisons of SWLS and CESD-20 scores of Study Group and Control Group.

	Study Group (n = 24)		Control Group (n = 24)		
	Mean	S.D.	Mean	S.D.	p-value
**SWLS**	19.4	6.8	27.0	4.5	<0.001
**CESD-20**	16.4	12.9	5.7	6.3	0.001
**CESD-20>15**	45.8%	(11/24)	8.3%	(2/24)	0.003

When analyzing the effects of various demographic and neurosensory deficit related parameters on life satisfaction in the Study Group, it was found that there were no correlations between the mean SWLS total score and the age groups (below 40 and 40 or above), gender of the patients, the nerve involved, time lapse of nerve injury, and the presence of pain or hyperaesthesia of the affected area (Table 4).

**Table 4:** Comparison of mean SWLS score and mean CESD-20 score by sample factors.

	Mean SWLS score (S.D.)		p-value	Mean CESD-20 score (S.D.)		p-value
**Age**			0.12			0.008
* Below 40*	21.5 (5.8)			10.2 (9.8)		
* 40 or above*	17.0 (7.3)			23.8 (12.5)		
**Gender**			0.56			0.45
* Male*	18.3 (5.5)			13.8 (15.7)		
* Female*	20.1 (7.5)			18.0 (11.2)		
**Nerve involved**			0.36			0.98
* IAN*	20.5 (8.1)			16.4 (12.6)		
* LN*	17.9 (4.3)			16.5 (14.0)		
**Time lapse of nerve injury**			0.80			0.12
* Less than 5 years (n = 16)*	17.9 (6.3)			17.9 (14.4)		
* 5 years or more (n = 8)*	22.4 (7.0)			13.5 (9.4)		
**Presence of pain**			0.84			0.38
* Yes*	18.7 (8.6)			22.7 (19.5)		
* No*	19.5 (6.7)			15.5 (12.1)		
**Presence of hyperaesthesia**			0.92			0.19
* Yes*	19.6 (7.6)			21.4 (14.4)		
* No*	19.3 (6.6)			13.9 (11.8)		

The effects of the various parameters on the CESD-20 total score in the Study Group were analyzed. It was found that patients with permanent neurosensory deficit of age 40 years or above were significantly related to higher CESD-score when compared to those who were younger than 40 years old. Gender of patients, the nerve involved, and the presence of pain or hyperaesthesia of the affected area were found to be not correlated with the CESD-20 total score (Table 4).

The Study Group patients with high CESD-20 score (16 or above) were compared with those with low CESD-20 score (15 or below). It was found that the patients in the high CESD-20 score group were significantly older than the low CESD-20 score group (mean age 45.1 years (S.D. 10.7 years) vs 35.0 years (S.D. 8.9 years), p = 0.019). There were no statistical differences between these two sub-groups in terms of gender, the nerve involved, and the presence of hyperaesthesia or pain of the affected area (Table 5).

**Table 5:** Comparison of sample factors of subjects with high and low CESD-20 score groups.

	Low CESD-20 Score Group(15 or below) (no. = 13)		High CESD-20 Score Group(16 or above) (no. = 11)		p-value
**Mean Age in years** (S.D.)	35.0 (8.9)		45.1 (10.7)		0.02
**Gender**					0.34
* Male*	46%		27%		
* Female*	54%		73%		
**Nerve involved**					0.63
* IAN*	54%		64%		
* LN*	46%		36%		
**Mean time lapse of nerve injury** (S.D.)	70.5 months (64.6 months)		38.5 months (44.2 months)		0.18
**Presence of pain** (% of sample)					0.44
* Yes*	8%		18%		
* No*	92%		82%		
**Presence of hyperaesthesia**(% of sample)					0.25
* Yes*	45%		23%		
* No*	55%		77%		

Based on the two primary outcomes SWLS and CESD-20 scores of the Study Group and the Control Group, the statistical power was calculated. For SWLS, the power was 99.4%, with a sample size to reach 90% power is 14. For CESD-20 score, the power was 94.7%, with a sample size to reach 90% power is 20.

## Discussion

The purpose of this study was to investigate the effects of permanent trigeminal neurosensory deficit after lower third molar surgery on life satisfaction and depressive symptom prevalence of the affected individuals. Based on the results of the study, we found that the life satisfaction of these individuals were significantly worse than those who did not suffer from the complication. There were also more depressive symptoms in these individuals. To our knowledge, this study is the first attempt in the literature to investigate the psychological impact on individuals suffering from trigeminal neurosensory disturbance as a consequence of lower third molar surgery.

Unlike motor deficit in motor nerve injury where direct measures of sensory changes are feasible, it is not easy to objectively measure sensory disturbance in trigeminal nerve injury after lower third molar surgery. While neurosensory deficit affects the individual’s subjective feeling, it is complicated by psychological effects on the sensation. In recent years patient-reported outcome measurement is becoming more popular to measure the effect of an intervention or complications after an intervention [9]. It represents the outcomes of an intervention or its complication from the patient’s perspective. Life satisfaction is one of the components of patient-reported outcome measurement that assesses the quality of an individual’s life on the basis of his or her own unique set of criteria. In a surgical complication like the neurosensory disturbance after lower third molar surgery, assessment of life satisfaction can reflect the subjective well being of the affected individual. It may serve as an efficient means of evaluating the effect of a surgical complication from the traditional objective assessment-based approach. SWLS is a well established tool to measure life satisfaction. Validated by self reports, peer reports, memory measures and clinical ratings, SWLS has been proven to indicate a relatively global and stable psychological attribute rather than a temporary judgment susceptible to fleeting influences, and it is suited for use with a wide range of age groups and applications. [10]In this study, the control group was composed of individuals who did not have such complication after oral surgery. The mean SWLS score in the control group was 27.0, which falls in the range of 26–30 or the range of *satisfied* reported in Pavot and Diener [4]. This level of satisfaction is similar to or slightly better than the general populations that were reported in the literature [3], [10], [11], [12]. In contrast, the mean SWLS score in individuals with neurosensory deficit after lower third molar surgery was 19.4. This figure falls below the “neutral point” of 20, the point at which the respondent is about equally satisfied and dissatisfied [3]. It was reported in other studies that life satisfaction could be drastically reduced after a severe injury like traumatic brain injury or spinal cord injury [13]–[16]. Post et al reported the mean SWLS score of individuals with spinal cord injury was 21.0, while Davis et al. reported the mean SWLS score of individuals suffered from traumatic brain injury was 21.3 [15], [16]. It was surprising that individuals with neurosensory deficit after lower third molar surgery perceived their life satisfaction to be even worse than those with more severe neurological deficit like spinal cord or traumatic brain injury. The mean SWLS score of these individuals was even comparable to those who suffered from moderate to high level of chronic pain after spinal cord injury, who had a mean SWLS score of 18.99 [13]. It reflected the negative effect on life satisfaction of an individual with neurosensory deficit after lower third molar surgery may be comparable to, if not worse than, some major traumatic injuries that are known to cause significant handicap.

Depressive symptoms may arise after an individual suffers from a complication of a surgery or from a disease [17]. Neurological disease or injuries are known to be associated with the development of clinical depression [18]–[20]. To our knowledge, neurosensory disturbances after maxillofacial surgical procedures have not been reported as a cause of the development of depression. The current study is the first attempt in the literature to show that patients with neurosensory deficit after lower third molar are having more depressive symptoms when compared to those without such complications. The CESD-20 score has been widely used as a screening tool for depression, with a higher score indicating more depressive symptoms of an individual. 85% of the people being diagnosed to suffer from depression by a psychiatrist were found to attain a high score on CESD-20. Moreover, the reliability and validity of CESD-20 have been well established among different populations, such as Chinese elderly. [21] It was also suggested that an individual with CESD-20 score of 16 or higher may need further assessment and evaluation for depression [8]. In this study, 50% of the study group participants scored a CESD-20 score of 16 or above, indicating a possible higher risk of these patients to develop clinical depression compared to the control group. It suggests that maxillofacial surgeons who see patients with neurosensory deficit after lower third molar surgery shall aware if they are suspected to have concurrent mood disorders, and may offer appropriate referral to these patients for evaluation and management if needed.

This study has not identified the major factors that affect the life satisfaction of patients with neurosensory deficit after lower third molar surgery. Among the patients in the study group, the unfavorable characteristics of the nerve injury like perceived severe numbness and the presence of pain or hyperaesthesia are not shown to correlate with the level of life satisfaction or depressive symptoms. This may be explained by the relatively small sample size of patients with pain or hyperaesthesia, as most of these patients with such bothersome symptoms would have undergone surgical repair of the damaged nerve. It was also noted time was not found to be a factor that allow psychological adjustment of the neurosensory deficit (i.e. improvement of life satisfaction or depression symptoms over time). Older age appears to be a predictor of worse CESD-20 score among these patients. Affected individuals with a CESD-20 score higher than or equal to the cut-off point of 16 are also significantly older. These findings suggest that younger patients may cope better psychologically with the neurosensory complication after third molar surgery, thereby consolidating the belief that if a lower third molar that is indicated for removal, it should be removed at a younger age.

## Conclusion

The results of this study suggests that individuals with permanent trigeminal neurosensory deficit after lower third molar surgery have worse life satisfaction and more depressive symptoms when compared to those who do not suffer from the surgical complication. Older age of an individual with permanent trigeminal neurosensory deficit after lower third molar surgery appears to be related to the development of more depressive symptoms.
